# Delay in Diagnosis and Treatment of Bone Sarcoma—Systematic Review

**DOI:** 10.3390/cancers17060981

**Published:** 2025-03-14

**Authors:** Dawid Ciechanowicz, Daniel Kotrych, Krzysztof Starszak, Piotr Prowans, Sławomir Zacha, Adam Kamiński, Adam Brodecki, Katarzyna Kotrych

**Affiliations:** 1 Department of Orthopaedics, Traumatology and Musculoskeletal Oncology, Pomeranian Medical University, 71-281 Szczecin, Poland; 2 Department of Children Orthopaedics and Musculoskeletal Oncology, Pomeranian Medical University, 71-281 Szczecin, Poland; 3 Department of Human Anatomy, Medical University of Silesia, 40-752 Katowice, Poland; 4 Department of Plastic, Endocrine and General Surgery, Pomeranian Medical University, 71-252 Szczecin, Poland; 5 West Pomeranian Oncology Center, Pomeranian Medical University, 71-730 Szczecin, Poland

**Keywords:** bone sarcoma, delay in diagnosis, symptoms, overall survival, treatment

## Abstract

Background: The problem of delay in diagnosis is significant in rare malignant tumors, such as bone sarcomas. The early detection of cancer and prompt treatment is crucial for outcomes and overall survival. A systematic review was conducted to determine the impact of delayed diagnosis on the overall survival and to identify factors that significantly affect this delay. Methods: A proportion of 36 out of 4585 articles met the inclusion criteria. Results: The average delay in diagnosis ranged from 7 weeks to 28 months. A younger age, female gender, extremity localization, and high-grade sarcomas were contributing factors to a shorter delay in diagnosis. The impact of long delays on the treatment outcomes and overall survival remains ambiguous. Although most of the analyzed papers showed worse survival in patients with a longer delay, these results were usually not statistically significant. Conclusion: Increasing oncological vigilance, educating physicians in the field of sarcoma diagnostics, and centralizing treatment are potential factors that can reduce the delay in diagnosis.

## Simple Summary

Bone sarcomas are classified as rare malignant tumors. For this reason, delay in the diagnosis of this group of patients still remain a significant problem. Therefore, the aim of this review is to analyze publications regarding the delay in the diagnosis of bone sarcoma and to attempt to determine the factors influencing this delay and to determine the consequences for patients. For this reason, we analyzed 4585 articles, of which 36 were included in the review. Due to different definitions of the term ‘delay in diagnosis’ and the high heterogeneity of sarcomas in the analyzed publications, a wide range in the duration of the delay was found—from 7 weeks to 28 months. Factors such as a younger age, female gender, location in the peripheral skeleton, and a histopathological diagnosis of osteosarcoma have been found to be associated with a shorter delay in diagnosis. A long delay was usually due to the lack of oncological vigilance of doctors and confusing the symptoms of sarcomas with other orthopedic diseases. Most of the analyzed papers showed worse survival in patients with a longer delay, but these results were usually not statistically significant. Improving education among physicians and increasing oncological vigilance among GPs and centralizing treatment may contribute to improving the earlier detection of bone sarcomas.

## 1. Introduction

Delay in the diagnosis and treatment of rare types of cancers such as bone sarcoma still remains an important problem. Primary malignant bone neoplasms (bone sarcoma) account for 0.2–0.5% of all malignant tumors worldwide. They are slightly more common in the pediatric population, where they account for 5–7% of all cancers [1,2,3]. Due to delays in accurate diagnosis and subsequent suboptimal or inappropriate treatment, patients suffering from rare cancers have a higher mortality rate compared to that of patients with common types of cancers, like breast or prostate cancer [4,5]. Despite the continuous development of new methods of treatment, including surgery, the overall 5-year survival rate in patients with bone sarcomas is about 60% [5,6,7]. Due to the lack of characteristic symptoms, low public awareness, and limited experience of healthcare workers with bone sarcoma, delay in diagnosis and treatment remains a common problem that has not improved significantly over the last few decades. This effect has also been compounded by the COVID-19 pandemic [8,9]. The early detection of the disease is a key element in oncological treatment and affects not only the overall survival of patients but also their quality of life [10]. Some authors indicate that, particularly in sarcoma patients, the larger the tumor size, the more extensive the surgery and the lower the chance for limb-sparing treatment. However, at the same time, it was demonstrated that the duration of symptoms did not correlate with the size of the tumor [11]. On the other hand, some studies have shown that a prolonged duration of symptoms is associated with a larger size of tumor and increased rate of metastasis, but the impact of delay on the overall outcome is still not clear [12]. To the best of our knowledge, there is a lack of systematic reviews analyzing in detail the causes and consequences of delay in the diagnosis of primary bone sarcomas. Therefore, the aim of this review is to identify the most common factors contributing to delays and the consequences of delayed diagnosis of bone sarcomas. We believe that the data collected will help us understand the causes of delay in diagnostics and help find ways to reduce this problem in the future.

## 2. Materials and Methods

**Figure 1:**
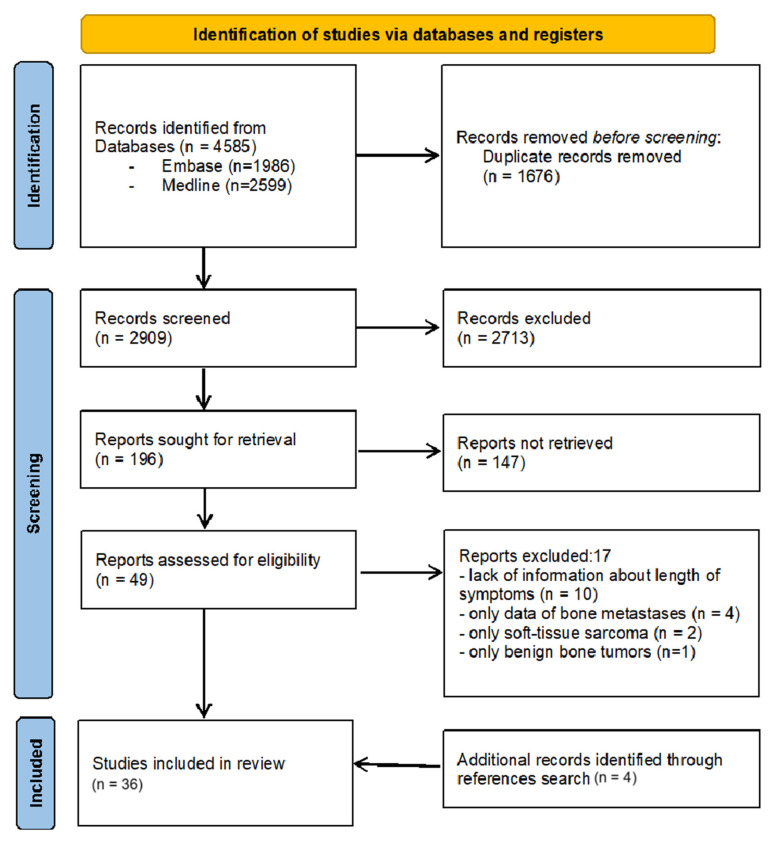
Flow-chart of the selection process for the studies included in the literature review.

The review was conducted in keeping with the Preferred Reporting Items for Systematic Reviews and Meta-Analyses (PRISMA) guidelines. The literature search was conducted in the MEDLINE/PubMed database and EMBASE/Ovid database. Additional potentially matching studies were pinpointed by cross-searching the referenced articles in terms of backward and forward citation. The search terms used were ‘bone sarcoma’ OR ‘bone cancer’ OR ‘bone tumor’ AND ‘delay in diagnosis’ OR ‘length of symptoms’. The search was restricted to the period January 1980–July 2024 with no restrictions regarding the study design. The search was conducted in August 2024. Three authors separately checked the published studies’ titles and thereafter checked their abstracts (D.C., K.S., and A.B.). The inclusion criteria were as follows: (1) The study had to include information on the duration of symptoms before the final diagnosis was made. (2) The study had to include patients with bone sarcoma. Only studies available in English and German were analyzed. In total, 4585 articles were identified. Of these articles, 1676 were removed due to duplicate reportage, and 2713 were excluded based on the titles and abstracts. The remaining 196 articles were accessed for the full text and screened for further assessment. Finally, 36 articles were included in the review (Figure 1). Data regarding factors influencing delay in diagnosis of bone sarcomas, such as age, gender, histopathological type of tumor, tumor location, and others, were analyzed and included in the review. Publications describing the impact of the duration of delay in diagnosis on overall survival, the incidence of metastases, and local recurrence in patients with bone sarcomas were also assessed. Due to the heterogeneity of inclusion criteria and methods, it was not possible to conduct a meta-analysis, so results were reported descriptively.

## 3. Results

The terms ‘delay in diagnosis’ or ‘length of symptoms’ are defined differently by the authors. In all of the included studies, the starting point of this time interval is the onset of the first symptoms reported by the patient. The end point, however, varies depending on the publication. Most often, it is the time of diagnosis—the initial diagnosis or histopathological diagnosis (20 studies). In contrast, Goyal et al. defined delay in diagnosis as the time from the onset of first symptoms to the day of performing biopsy, while Schurr et al. defined delay as the time until the initiation of treatment. In the studies included in the review, the average delay in diagnosis ranged from 7 weeks to 28 months [Table 1]. Differences in the length of the delay depend on the histological type of the bone sarcoma, its location, and the country in which the study was conducted. Factors influencing the length of the delay are analyzed later in the text. Some publications (*n* = 8) divided delay in diagnosis into patient-related (PRD), defined as the time from the first symptoms to the time of reporting to a doctor, and doctor-related (DRD), defined as the period from first doctor appointment to the end point—final diagnosis. In the analyzed publications, a trend can be observed indicating that patients report symptoms of bone neoplasms to a doctor relatively quickly, making the patient-related delay 1–3 months. Goedhart et al. showed that DRD is more than twice as long as PRD for osteosarcoma (100 vs. 44.8 days) and Ewing sarcoma (130.6 vs. 41.0 days) [13]. Also, Schurr showed a longer DRD for primary bone tumors—19 vs. 10.5 weeks [14]. Pan et al. showed a longer PRD compared to DRD (10 vs. 3 weeks) in patients with osteosarcoma around the knee [15]. It is worth noting in the above study that all patients had an X-ray examination performed during the first visit, which allowed the time from seeing a doctor to making a diagnosis to be shortened.

### 3.1. Variables Influencing Delay in Diagnosis

#### 3.1.1. Gender

Three studies included in the review examined the effect of gender in diagnostic delay. Sneppen et al. and Smith et al. did not indicate that gender had a significant impact on the delay [16,29]. Wide et al., in their study of Ewing sarcoma located in the ribs, showed a longer delay for men compared to women (13 weeks vs. 7 weeks), but without statistical significance [26]. However, Pollock, in a study in the pediatric population, showed that female gender is associated with a shorter diagnosis delay in the Ewing sarcoma group. The girls had a decreased lag time (time from first symptoms to diagnosis) for Ewing sarcoma and osteosarcoma compared to that of the boys (*p* = 0.02) [17]. In another study on the adult population, Schnurr et al. showed a longer patient-related delay in women as compared to males [14].

#### 3.1.2. Age

The included studies indicated a greater delay in diagnosis in adult patients. Sneppen et al. showed that patients with osteosarcoma over 20 years had a longer delay compared to younger patients—4.7 vs. 9.1 months (*p* < 0.001) [16]. Similar results were reported by Nandra et al., where the cut-off point was set at 22 years—the younger patients showed a shorter delay compared to that of the older group: 12 vs. 28 weeks (*p* < 0.05) [34]. Goyal et al. showed that, in the case of Ewing sarcoma, patients under 12 years of age have a shorter delay: 0.4 vs. 2.4 months [20]. However, Guerra et al. did not observe a similar relationship in a group of 365 patients with Ewing sarcoma and osteosarcoma under 30 years of age [22]. Contrastingly, older age has been found to have a significant prognostic impact (*p* = 0.010) on the length of the symptom interval in a study published by Schnurr et al. An age between 30 and 60 years (HR = 1.384 (95%-CI: 0.972–1.970), *p* = 0.072) and an age over 60 years (HR = 1.740 (1.209–2.505), *p* = 0.003) significantly corresponded to a shortened symptom interval when compared to the level of those aged under 30 years [14]. But, in contrast, the physician needed significantly more time for patients younger than 30 years in comparison to older patients before initiating the first specific therapy. This was also confirmed by Lawrenz et al., in their study, where they reported that every additional year of age was associated with a 1.3-week-longer total interval of symptoms [38].

#### 3.1.3. Tumor Localization

Some studies indicate a shorter delay in diagnosis in the case of tumors located in the limbs compared to those in the axial skeleton. Goyal et al. indicated a longer doctor-related delay in the case of axially located tumors: 4.7 vs. 1.4 months (*p* = 0.002) [20]. Snappen et al. showed a shorter delay time for osteosarcoma located in the limbs compared to the trunk, 5.8 vs. 9.3 months, with no significant difference (*p* > 0.05). However, it showed a shorter delay time in patients with Ewing sarcoma located in the upper limbs compared to the lower limbs: 2.6 vs. 14.3 months (*p* = 0.01) [16]. Rougraff et al., in their study, did not show a significant statistical difference when comparing the delay in bone sarcomas located in the limbs and the axial skeleton [24]. As reported by Gerrand et al., patients with non-extremity bone sarcomas were more likely to be diagnosed after an Emergency Department visit compared to after GP referral, compared to extremity bone sarcomas. In addition, patients presenting by emergency routes more often had metastases and had less than 1 year survival [48].

#### 3.1.4. Type of Bone Tumor

Pollock, in his study, showed that bone sarcomas (OS and ES) have the longest delay in diagnosis compared to other childhood cancers (56–72 days vs. 21–49 days) [17]. Osteosarcoma has a shorter delay in diagnosis compared to that of Ewing sarcoma. Ewing sarcoma is often more difficult to detect because it tends to arise in axial sites, such as the pelvis, whereas osteosarcoma arises in the more readily observed long bones of the extremities. This was confirmed by Widhe et al. in a study that showed a longer doctor’s delay for Ewing sarcoma than for osteosarcoma (19 weeks and 9 weeks, respectively; *p* < 0.0001) [49]. A similar trend was reported by Guerra et al. in their study, where the average time until diagnosis was 21.1 weeks for osteosarcoma, while for Ewing sarcoma, the average time until diagnosis was 32.4 weeks [22]. Goethart et al., in their study, showed that, in patients with chondrosarcoma, the mean total delay is significantly longer than that for osteosarcoma and Ewing sarcoma (688 vs. 163.3 vs. 160.2 days, *p* < 0.01) [13]. The patients diagnosed with chondrosarcoma had a longer delay compared to patients with osteosarcoma also in the study by Lawrenz et al.: 52 vs. 12 weeks (*p* < 0.001). Moreover, the authors showed that intermediate-grade tumors had a markedly longer median delay in diagnosis compared with that for high-grade tumors (52 vs. 12 weeks, *p* < 0.001) [38]. Rougraff also showed a longer duration of symptoms in low-grade chondrosarcoma (14.6 months), compared to that in high-grade sarcoma (9.8 months), Ewing sarcoma (6.7 months), and osteosarcoma (2.8 months) [24].

#### 3.1.5. Others

The patients presenting continuous and increasing pain showed a shorter delay time compared to the patients presenting intermittent pain—3.0 vs. 12.6 months in the Ewing sarcoma group. Also, patients with swelling as one of the first symptoms reached the oncology center in a shorter time, both in the case of osteosarcoma (4.6 vs. 7 months) and Ewing sarcoma (3.2 vs. 10.1 months) [16].

The type of health professional initially consulted also made a difference, with the median total delay in diagnosis for those presenting to a GP being 4.3 vs. 2.8 months for A&E presenters [20]. The median interval between the first presentation to a health professional and seeing a specialist was 0.9 months if presenting to a GP compared with 0.4 months if presenting to A&E. The patients with a trauma history (*n* = 17) sought medical advice earlier than those without such a history (*n* = 34), the mean duration of symptoms to first medical consultation being 20 versus 62 days, respectively (*p* = 0.025) [25].

Smith et al., in their study, put forward the thesis that in obese people, due to the greater amount of adipose tissue, there is a greater chance of missing swelling or palpable lumps in the limbs, which may result in a longer delay in diagnosis. However, the study results did not indicate that obese patients had a longer delay compared to patients with a normal BMI [29].

In the study conducted by Nandra et al., they showed that the median duration of symptoms varied most significantly with the grade of the tumor. The patients with high-grade sarcoma had a significantly shorter duration of symptoms than those with lower-grade tumors and this was most marked for bone tumors [34].

Postl et al. showed a significantly longer delay in the diagnosis of bone sarcoma in pregnant women compared to a non-pregnant group—8.0 vs. 6.1 months (*p* = 0.039) [35].

Information about a prior tumor was associated with a significantly shorter patient delay as compared to when this information was missing, i.e., in the case of metastases and unknown primary tumor or primary bone tumor [14].

### 3.2. Why Does Delay in Diagnosis Occur?

#### 3.2.1. Lack of Oncological Vigilance and Lack of Radiological Examinations

Bone sarcomas are a group of rare tumors, which means that most doctors may not see any patients with this condition or only a few cases during their entire career. This means that patients presenting with pain in the musculoskeletal system are treated for other more common diseases. Moreover, in such cases, a small number of doctors decide to perform a radiological examination if there is no injury. This was confirmed by Goyal et al., where, in the group of patients with bone sarcomas who reported to a physician with typical symptoms, only 61% of the patients had a radiological examination [20]. Similar results were presented by Kotrych et al., where, after the first medical consultation, approximately 60% of the patients had an X-ray examination, of which only approximately 47% were further referred to an oncology center [45]. According to George et al., patients with bone sarcomas visited physicians, on average, 1.5 months after observing the first symptoms of disease, of which 88% presented symptoms (so-called red flags) that suggested the need to perform radiological examinations. However, this procedure was performed in 54% of the patients in the study group [30]. According to Wrutz, about 44% of the pelvic sarcomas were not accurately diagnosed for at least one month from the time that the patient first visited a physician for an evaluation of symptoms. The initial radiographs of eight of the thirty patients were misinterpreted by general practitioners, radiologists, or orthopedic surgeons as showing normal findings, a stress fracture, or degenerative arthritis. In at least nine of the thirty patients, the symptoms were presumed to be the result of a disease of the lumbar spine; therefore, the initial diagnostic evaluation of these patients was focused on the lumbar spine rather than on the pelvis. Osteosarcoma was detected in 72% of cases after X-ray examination [19]. Moreover, according to Yang et al., in their study analyzing the histories of patients with osteosarcoma, a minimum of two medical consultations were necessary before bone sarcoma was suspected, but in 31% of cases, three consultations were needed [25]. Interestingly, according to Smith et al., the publication of recommendations for the diagnosis and treatment of bone sarcomas has not reduced the delay in diagnosis in the UK over the years [29].

#### 3.2.2. Misdiagnosis

All doctors know the following saying perfectly well: “when you hear hoofbeats, think horses not zebras”. It is important, however, not to completely forget about zebras. Most patients with bone sarcomas are initially treated for common orthopedic diseases. This assumption is supported by Widhe et al. in their study, where a high percentage of false diagnoses of patients younger than 30 years with bone tumors were reported. About 31% of patients with osteosarcoma in the limbs were diagnosed with tendinitis and about 12% with uncertain pain [48]. Kotrych et al., however, states that, before referral to an oncology center, patients were most often treated for trauma (25%), sciatica (12.5%), arthritis (12.5%), and enthesopathy (6.25%) [45]. However, according to Wrutz, in the case of bone sarcomas located in the pelvis, the incorrect diagnoses included a herniated lumbar disc, spinal stenosis, spondylolisthesis, tendinitis, bursitis, an inguinal hernia, a stress fracture, a pilonidal cyst, a recurrent urinary tract infection, and degenerative arthritis of the spine, hip, and knee [19]. Patients with Ewing sarcoma were most often misdiagnosed and treated for acute osteomyelitis, especially in the pediatric population [50]. However, incorrect diagnosis does not concern only GPs and orthopedists. According to Kim et al., in a group of patients with osteosarcoma, histopathological or radiological misdiagnosis was recorded in approximately 54% of cases [27]. According to Biscalglia, radiological misdiagnosis occurred in half of the cases of bone sarcomas in the foot [18]. It is worth noting here that a radiograph first interpreted as normal, as was the case for 35% of the patients in the study conducted by Widhe et al., led to a significantly prolonged doctor’s delay [28].

### 3.3. Consequences of Delay in Diagnosis

According to Grimer et al., for every 1 cm increase in the size of a soft tissue sarcoma at diagnosis, there is a 3% to 5% worsening of the chance of cure, although the effect of size on prognosis is less marked for bone tumors [51]. A total of 14 studies included in this review tried to answer the question of whether a longer period of delay in diagnosis significantly affects the survival of patients [13,16,19,20,23,24,25,28,33,34,38,40,41,43]. Eleven of them did not show a significant relationship between a longer delay and overall survival. However, Widhe et al. showed that, in patients with chondrosarcoma, a total delay longer than 8 months carried a significantly higher risk of tumor-related death. However, these data refer to sarcomas located only in the chest [28]. In another study, Hu et al., in a group of over 180 patients with osteosarcoma, showed a tendency towards worse overall survival for patients with symptom delays of longer than 60 days. However, they were unable to find a positive correlation in the multivariate analysis between a prolonged delay and overall survival [40]. Goedhart, in his study of patients with high-grade bone sarcoma, showed a lower 5-year overall survival when the delay was more than 42 days: for osteosarcoma, 58.2% vs. 76.9%; for chondrosarcoma, 46.2% vs. 83.3%; and for Ewing sarcoma, 39.4% vs. 80%. However, these differences were not statistically significant [13]. Furthermore, Petrilli et al. did not show that a longer delay in diagnosis was associated with larger tumor size, the presence of metastases, or overall patient survival. However, tumors over > 12 cm had a negative impact on the survival of osteosarcoma patients [23]. Rougraff et al., in a group of 624 patients with bone sarcomas, showed that a greater duration of symptoms did not correlate with lower survival or continuous disease-free survival [24]. Yoshida et al. showed that a delay in consultation with a specialist in pediatric patients with osteosarcoma of more than 4 weeks may have increased the risk of subsequent metastases that were not detected at the time of diagnosis [44]. Among other consequences of delay in diagnosis, Bielack et al. mention the presence of metastases. They showed that patients with primary metastatic osteosarcoma were more likely to have a long history of symptoms [52]. Similar results were obtained in patients with Ewing sarcoma, where it was proved that a diagnostic interval < 2 months was associated with an increased likelihood of metastases at diagnosis [53]. However, Yang et al. showed that, in a group of pediatric patients (*n* = 51) with osteosarcoma presenting with metastatic disease, they had a similar duration of symptoms as those with non-metastatic disease—42 days versus 50 days [25]. On the other hand, Altıntaş et al. showed that patients with a diagnostic delay of 3 months or less had a higher rate of recurrence and mortality [46]. In contrast, Lawrenz et al., in their study, where they analyzed data from about 1792 patients with bone sarcoma, showed that a longer duration of symptoms was associated with longer survival [38]. This suggests that in low-grade sarcoma, where the duration of symptoms is longer compared to that with high-grade sarcoma, the prognosis for overall survival is better.

## 4. Discussion

The primary objective of the review was to determine the consequences of delays in the diagnosis of bone sarcomas, but the analyzed articles do not clearly indicate that a longer delay negatively affects the overall outcome. However, as indicated by the study by R.D. Neal, diagnostic delays in cancer do matter, but it is difficult to quantify their impact on survival or mortality [54]. This is also confirmed by our review, where most of the analyzed papers showed worse survival in patients with a longer delay, but these results were not always statistically significant [13,16,19,20,23,24,25,28,33,34,38,40,41,43]. Among other consequences, Mesko et al. showed that in 81% of cases, a delay in diagnosis was part of the complaint, and a further 7% were about misdiagnosis and 11% about unnecessary amputation. Primary care doctors and orthopedic specialists were the most common defendants in delay in diagnosis cases [55]. Another aim of the review was to identify factors that significantly influenced delay in diagnosis in bone sarcoma patients. Factors such as a younger age, female gender, and location in the peripheral skeleton have been found to be associated with a shorter delay in diagnosis [3,16,17,20,24,26]. In addition, osteosarcoma and Ewing sarcoma have been reported to exhibit a shorter delay time compared to that with chondrosarcoma [13,38]. Low-grade sarcoma has also been shown to be associated with a longer delay time [24,38].

Although the studies analyzed in this systematic review do not clearly indicate the influence of a longer delay in diagnosis on the overall survival, one of our main goals is to emphasize that early diagnosis and early appropriate treatment are important in the case of bone sarcomas. This is also a subject of consideration in the literature by researchers dealing with cancers localized outside of the musculoskeletal system. A term that is often repeated in prevention programs, public health, and medical education is “oncological vigilance” [34]. Yoshida et al. believe that the most important thing for the patient is an early examination and quick referral to a specialist center [44]. The topic of quick referral to specialist clinics is also discussed in the publication by Goyal et al., who point out its validity, even in the absence of confirmation of the diagnosis, or ultimately, no effect on the time of initial diagnostics [20]. Self-examination, observation, and heeding symptoms can contribute to the early detection of cancer, influencing the speed of treatment implementation. Despite reported symptoms associated with the musculoskeletal system, primary care physicians, who are the “gateway” to specialist treatment, contribute to delays in diagnosis through insufficient involvement in the diagnostic process. Primary care physicians should be substantively prepared to conduct a subjective and physical examination, including determining the location of the tumor and its macrostructure [22,25]. Schnurr et al. draw attention to the insufficient and less up-to-date knowledge of musculoskeletal oncology among physicians working in rural areas. An additional problem is also the limited access of patients to specialists in less urbanized areas [14]. Alarming symptoms—pain or swelling correlated with a history that may suggest a neoplastic component—should always result in expanding the diagnostics to include imaging tests such as X-rays and issuing a referral to a specialist in orthopedics and traumatology. Goedhart et al. emphasized that the increase in the diagnosis of bone cancer could be influenced by quickly referring patients for the simplest radiological examination, especially if the pain is persistent and long-lasting (over 6 weeks), and the history of trauma is negative [13]. Biscalglia et al. postulated the need for imaging diagnostics in the case of idiopathic pain lasting over 10 days [18]. This type of procedure should be considered the gold standard, and the potential overrepresentation of X-rays and specialist consultations performed without determining the palpable pathologies is morally acceptable in the face of the threat of bone cancer. The study being carried out and the extended diagnostic pathway should result from appropriate education on bone cancer and social programs conducted on a large scale, which are still lacking. In this context, George et al. raise the problem of potentially too much educational involvement of patients in the field of musculoskeletal oncology, which may influence the arousal of fear of cancer, consequently generating extra costs for additional tests, forcing this responsibility on doctors, while their justification may be debatable at that moment in time [30]. Medical education, also at the academic level, is too limited on the subject of musculoskeletal oncology, probably due to the rare occurrence of these diseases in the general population. This is a mistake, considering the statistics. To avoid delays in diagnosis, attention should be paid to symptoms—local pain, swelling, and, less frequently, which is characteristic of advanced stages of cancer, pathological fractures [9,30,45,51]. In the case of Ewing sarcoma, fever may be an important aspect of differential diagnosis with other types of cancer [16,50]. Potential trauma, often not ruled out by the patient in the subjective examination, is a limitation of the doctor, focused primarily on the most obvious cause of pain, forgetting about the possibility of cancer development [19,30,45]. Patients who downplay symptoms, dissimulate, and deny the possibility of cancer despite worrying symptoms are also problematic in early diagnosis. However, this is a highly prevalent group of patients in the entire patient population, regardless of the disease profile. The preparation of standards and guidelines in the scope of basic diagnostics, taking into account oncological vigilance even at the stage of primary care, seems to be a solution that can greatly contribute to accelerating the diagnostic process and increasing the percentage of early detections of neoplastic diseases in the musculoskeletal system. The next important step in improving the diagnosis pathway (especially regarding doctor-related delay) of patients with bone sarcomas is treatment in centers dedicated to patients with bone sarcomas. Centralizing care at sarcoma centers with a multidisciplinary team improves the diagnostic interval [56,57,58,59]. One of the reasons for this is the shorter waiting time for the results of histopathological examinations [45]. Patients also receive appropriate imaging for tumor staging [59]. In addition, treatment in reference centers reduces the risk of so-called ‘whoops surgery’ (Unplanned Sarcoma Resections), which reduces the risk of local recurrence of sarcoma [60]. In Poland, each patient, after consultation at the sarcoma center, receives a diagnosis and oncological treatment card (DILO), which allows them to receive full radiological diagnostics in less than 2 weeks [45,61]. Some countries provide a free national consultation service in order to improve the quality of diagnosis and to avoid the risk of delay in diagnosis. For example, in Japan, an online consultation service and a cancer image reference database (NCC-CIR) was created. According to the authors, musculoskeletal neoplasms were one of the most common reasons for consultation [62]. This gave physicians the opportunity to quickly consult on rare diseases, which helped shorten the time to diagnosis.

## 5. Conclusions

Available publications are inconsistent regarding the influence of delay in diagnosis on overall survival, local recurrent rates, and metastasis rates in patients with bone sarcomas. The duration of symptoms in patients with bone sarcomas before treatment indicates an existing problem with diagnostic delay. This is influenced by some factors, such as age, gender, location, or the histopathological type of the sarcoma. However, improving education among physicians, increasing oncological vigilance among GPs, and centralizing treatment may contribute to improving the earlier detection of bone sarcomas.

## Figures and Tables

**Table 1:** Characteristics of the studies included in the systematic review. Abbreviations: IS—initial symptoms, OC—oncology center, HS—histopathological diagnosis, D—diagnosis, B—biopsy, T—treatment, FMC—first medical consultation, PRD—patient-related delay, DRD—doctor-related delay, LG—low grade, HG—high grade.

First Author	Country, Year	Patients, *n*	Age Interval [Years]	Delay Definition	Tumor Type	General Delay [Mean]	Comments
Sneppen [16]	Denmark, 1984	124	17–28	IS—OC	Osteosarcoma Ewing sarcoma	6.4 months 9.6 months	None
Pollock [17]	USA, 1991	528	1–29	IS-D	Osteosarcoma Ewing sarcoma	11.6 (median 8) weeks 20.9 (median 10) weeks	None
Biscaglia [18]	Italy, 1998	12	17–64	IS—HS	Osteosarcoma	28 months	Only tumors in feet
Wurtz [19]	USA, 1999	68	8–82	IS-HS	Bone sarcomas	10 months	Only tumors in pelvis
Goyal [20]	UK, 2004	103	4–22	IS-B	Osteosarcoma Ewing sarcoma	3.4 months 5.7 months	None
Simpson [21]	UK, 2005	19	3–57	IS-HS	Ewing sarcoma	PRD—6 months DRD—5 weeks	Only tumors in upper limb
Guerra [22]	Brazil, 2006	365	0.2–30 1–30	IS-HS	Osteosarcoma Ewing sarcoma	21.2 weeks 32.4 weeks	None
Petrilli [23]	Brazil, 2006	225	2.4–24.5	IS-D	Osteosarcoma	18.4 weeks	None
Rougraff [24]	USA, 2007	242	NA	IS-D	Osteosarcoma Ewing sarcoma Chondrosarcoma LG Chondrosarcoma HG	2.8 (median 2.3) months 6.7 (median 3.5) months 14.6 (median 6) months 9.8 (median 4.7) months	None
Schnurr [14]	Germany, 2008	265	5–87	IS-T	Primary bone tumors	29.5 weeks	None
Yang [25]	China, 2009	51	3–20	IS—FMC	Osteosarcoma	8.7 weeks	None
Widhe [26]	Sweden, 2009	26	6–26	IS—D	Ewing sarcoma	PRD—2.5 months DRD—3 months	Only tumors in chest
Kim [27]	Korea, 2009	26	4–67	IS-OC	Osteosarcoma HG	DRD—10.5 months	None
Pan [15]	Malaysia, 2010	30	9–34	IS-HS	Osteosarcoma	17 weeks	None
Widhe [28]	Sweden, 2011	106	Mean 57	IS-D	Chondrosarcoma	8 months	Only tumors in chest
Smith [29]	UK, 2011	2568	Median 25	Duration of symptoms	Bone sarcoma	median 16 weeks	None
George [30]	UK, 2012	41	17–86	IS-D	Bone sarcoma	PRD—1.5 months DRD—3.9 months	None
Brotzmann [31]	Switzerland, 2013	32	9.8–72.9	IS-HS	Osteosarcoma	15 months	Only tumors in feet
Young [32]	Scotland, 2013	57	10–78	IS-T	Primary bone tumor	5–10 months	Only tumors in feet
Brasme [33]	France, 2014	436	9–15	IS-B	Ewing sarcoma	10 weeks	None
Nandra [34]	UK, 2015	2668	Median 22	IS-FMC	Bone sarcoma	16 weeks	None
Postl [35]	Germany, 2015	240	16–45	IS-D	Bone sarcoma	8 months	None
Goedhart [13]	The Netherlands, 2016	102	5–89	IS-HS	Osteosarcoma Ewing sarcoma Chondrosarcoma	23.3 weeks 22.9 weeks 98.3	None
Yang [36]	UK, 2017	55	Median 37	IS-D	Bone sarcoma	52 weeks	Only tumors in feet
Chen [37]	USA, 2017	30	0.3–24	IS-D	Bone sarcoma	12.4 weeks	None
Lawrenzn [38]	UK, 2018	1807	Mean 30.7	IS-OC	Bone sarcoma Osteosarcoma Chondrosarcoma	16 weeks 12 weeks 52 weeks	None
Domett [39]	UK, 2019	7	15–18	IS-D	Bone sarcoma	12.3 weeks	None
Hu [40]	China, 2019	182	12–59	IS-D	Osteosarcoma	9.7 weeks	Tumors located around knee
Bilal [41]	Lebanon, 2019	38	1–18	IS-D	Osteosarcoma	7 weeks	None
Belmant [42]	Brazil, 2019	1868	0–29	OC-T	Bone sarcoma	26 days	None
Letaief [43]	Tunisia, 2020	85	1–62	IS-OC	Osteosarcoma	14.7 weeks	None
Yoshida [44]	UK, 2021	250	4–16	IS-OC	Osteosarcoma	8 weeks	None
Kotrych [9]	Poland, 2023	87	16–90	IS-OC	Bone sarcoma	7–10 months	None
Kotrych [45]	Poland, 2023	32	18–87	IS-OC	Osteosarcoma Chondrosarcoma Giant cell tumor	6 months 8 months 5.5 months	
Altıntaş [46]	Turkey, 2024	59	6–18	IS-D	Osteosarcoma	3 months	None
Jenkins [47]	USA, 2024	288 (25)	NA	IS-OC	Bone sarcoma	5 months	Only tumors in feet

## Data Availability

Not applicable.
